# *catena*-Poly[[[triaqua­cobalt(II)]-μ-10-methyl­phenothia­zine-3,7-dicarboxyl­ato] monohydrate]

**DOI:** 10.1107/S1600536812009580

**Published:** 2012-03-10

**Authors:** Yun-Xia Hu, Yan Zhou, Fang-Ming Wang, Wen-Wei Zhang

**Affiliations:** a Xinjiang Laboratory of Phase Transitions and Microstructures of Condensed Matter, College of Chemistry and Biological Sciences, Yili Normal University, Yili, Xinjiang 835000, People’s Republic of China; b State Key Laboratory of Coordination Chemistry, School of Chemistry and Chemical Engineering, Nanjing University, Nanjing 210093, People’s Republic of China; c School of Biology and Chemical Engineering, Jiangsu University of Science and Technology, Zhenjiang 212003, People’s Republic of China

## Abstract

The polymeric title compound, {[Co(C_15_H_9_NO_4_S)(H_2_O)_3_]·H_2_O}_*n*_, consists of chains along [001] made up from Co^2+^ ions bridged by 10-methyl­phenothia­zine-3,7-dicarboxyl­ate anions. The Co^2+^ ion, coordinated by three O atoms from two different carboxyl­ate groups and three water mol­ecules, displays a distorted octa­hedral environment. In the crystal, π–π inter­chain inter­actions, with centroid–centroid distances of 3.656 (2) and 3.669 (2) Å between the benzene rings of the ligands, assemble the chains into sheets parallel to (100). O—H⋯O hydrogen-bonding inter­actions between the coordinating water mol­ecules and carboxyl­ate O atoms link the sheets into a three-dimensional network.

## Related literature

For background to phenothia­zine as a pharmacophore, see: Albery *et al.* (1979 ▶); Tsakovska & Pajeva (2006 ▶). For compounds with organic framework structures and with electro-optic or electronic properties, see: Chakraborty *et al.* (2005 ▶); Cho *et al.* (2006 ▶); Park *et al.* (2008 ▶); Krämer *et al.* (2001 ▶); Zhang *et al.* (2007 ▶). For structure elucidation, see: Spek (2009 ▶). 
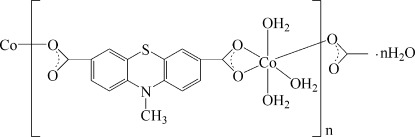


## Experimental

### 

#### Crystal data

[Co(C_15_H_9_NO_4_S)(H_2_O)_3_]·H_2_O
*M* *_r_* = 430.30
Orthorhombic, 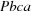

*a* = 15.3105 (8) Å
*b* = 7.2983 (4) Å
*c* = 29.5679 (15) Å
*V* = 3303.9 (3) Å^3^
*Z* = 8
Mo *K*α radiation
μ = 1.21 mm^−1^
*T* = 291 K
0.30 × 0.26 × 0.24 mm

#### Data collection

Bruker SMART CCD diffractometer
Absorption correction: multi-scan (*SADABS*; Bruker, 2000 ▶) *T*_min_ = 0.703, *T*_max_ = 0.759
16812 measured reflections
3236 independent reflections
2650 reflections with *I* > 2σ(*I*)
*R*_int_ = 0.049

#### Refinement

*R*[*F* ^2^ > 2σ(*F* ^2^)] = 0.043
*wR*(*F* ^2^) = 0.104
*S* = 1.08
3236 reflections
236 parameters
H-atom parameters constrained
Δρ_max_ = 0.62 e Å^−3^
Δρ_min_ = −0.52 e Å^−3^

### 

Data collection: *SMART* (Bruker, 2000 ▶); cell refinement: *SAINT* (Bruker, 2000 ▶); data reduction: *SAINT*; program(s) used to solve structure: *SHELXTL* (Sheldrick, 2008 ▶); program(s) used to refine structure: *SHELXTL*; molecular graphics: *SHELXTL* and *DIAMOND* (Brandenburg, 2006 ▶); software used to prepare material for publication: *SHELXTL*.

## Supplementary Material

Crystal structure: contains datablock(s) I, global. DOI: 10.1107/S1600536812009580/wm2597sup1.cif

Structure factors: contains datablock(s) I. DOI: 10.1107/S1600536812009580/wm2597Isup2.hkl

Additional supplementary materials:  crystallographic information; 3D view; checkCIF report

## Figures and Tables

**Table 1:** Selected bond lengths (Å)

Co1—O4^i^	2.0523 (19)
Co1—O5	2.074 (2)
Co1—O7	2.087 (2)
Co1—O6	2.114 (2)
Co1—O2	2.1581 (19)
Co1—O1	2.1661 (19)

Symmetry code: (i) 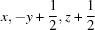.

**Table 2:** Hydrogen-bond geometry (Å, °)

*D*—H⋯*A*	*D*—H	H⋯*A*	*D*⋯*A*	*D*—H⋯*A*
O5—H5*X*⋯O8^ii^	0.85	1.83	2.678 (4)	180
O5—H5*Y*⋯O4^iii^	0.85	2.16	2.879 (3)	142
O6—H6*X*⋯O1^iv^	0.85	1.91	2.748 (3)	169
O6—H6*Y*⋯O8	0.85	2.43	3.149 (4)	143
O7—H7*X*⋯O2^v^	0.85	2.00	2.826 (3)	163
O7—H7*Y*⋯O3^i^	0.85	1.88	2.615 (3)	144
O8—H8*X*⋯O3^i^	0.85	2.01	2.808 (4)	156
O8—H8*Y*⋯O2^vi^	0.85	2.09	2.760 (3)	135

Symmetry codes: (i) 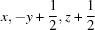; (ii) 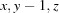; (iii) 
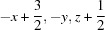
; (iv) 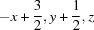; (v) 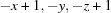; (vi) 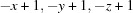.

**Fig. 1.**
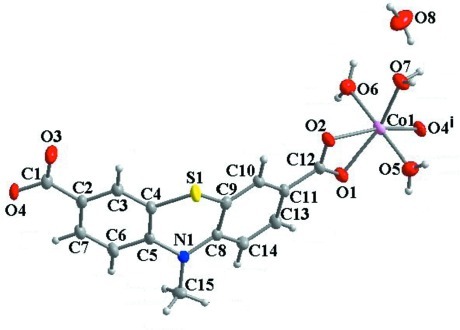
ORTEP plot of the title compound showing the local coordination environment of Co2+ with displacement ellipsoids at the 50% probability levels and H atoms shown as small spheres of arbitrary radius. [Symmetry code: i = x, 0.5 - y, 0.5 + z.]

**Fig. 2.**
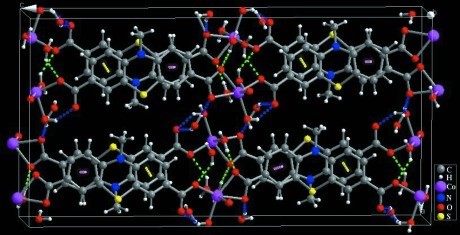
The crystal packing diagram showing the 3-dimensional network. Inter-chain (blue dotted lines), intra-chain (bright green dotted lines), hydrogen bonds and inter-chain π—π interactions (yellow and rose dashed lines) are displayed.

## supplementary crystallographic information

### Comment

Phenothiazine, an intriguing type of biologically and pharmaceutically active heterocyclic compound well known as a pharmacophore in tranquilizers, antituberculosis agents, anti-tumor agents, bactericides, *etc*. (Albery *et al.*, 1979; Tsakovska & Pajeva, 2006), is now widely studied as an electron donor component and electrically conducting charge-transfer composite on account of its unique electro-optic properties in materials science (Chakraborty *et al.*, 2005; Cho *et al.*, 2006; Park *et al.*, 2008). Previous studies involving this compound had more emphasis on the large π-electron conjugated system (Krämer *et al.*, 2001; Zhang *et al.*, 2007); however, less work is reported on the construction of metal-organic frameworks using it as a building block. Here we employed the 10-methyl-10*H*-phenothiazine-3,7-dicarboxylate (MPTD) anion as a ligand to crystallize the title complex.

The title compound, {[Co(C_15_H_9_NO_4_S)(H_2_O)_3_]^.^H_2_O}, consists of a three-dimensional supramolecular network built up from coordination bonds, hydrogen bonds, and π—π interactions. As shown in Fig. 1, the Co^2+^ ion has a slightly distorted octahedral coordination environment formed by three O atoms from two different carboxylate ligands and three O atoms from three coordinated water molecules. Each MPTD ligand bridges two Co atoms *via* two carboxylate groups in a monodentate and a bidentate coordination mode into a one-dimensional zigzag chain parallel to [001]. These chains are assembled in an antiparallel manner into two-dimensional sheets parallel (100) based on strong interchain π—π interactions between the ligands [centroid-centroid distance = 3.656, 3.669 Å]. The sheets are further connected to form a three-dimensional supramolecular network (Fig. 2) *via* interlayer O—H···O hydrogen bond interactions. A *PLATON* calculation (Spek, 2009) shows that the structure has 13.6% solvent accessible voids when the coordinated and lattice water molecules are neglected. The resulting framework structure contains channels with approximate dimensions of 2.9×4.9 Å^2^ and 1.9×1.9 Å^2^ along [010] and [001], respectively. All the lattice water molecules and the coordinating water molecules are situated in these channels and are involved in the above extensive interlayer and intralayer H-bonding.

### Experimental

The educt 10-methyl-10*H*-phenothiazine-3,7-dicarboxylic acid used to construct the title compound {[Co(C_15_H_9_NO_4_S)(H_2_O)_3_]^.^H_2_O} was prepared by oxidation of 10-methyl-10*H*-phenothiazine-3,7-dicarbaldehyde using silver nitrate as oxidant in an alkaline medium. 10-Methyl-10*H*-phenothiazine-3,7-dicarbaldehyde (0.6417 g, 2.38 mmol) (Cho *et al.*, 2006) was dissolved in 35 ml solution of KOH (10.0 g, 0.178 mol), then a 5 ml solution of AgNO_3_ (1.3 g, 7.65 mmol) was added slowly. The mixture was filtered after heating at 103 K overnight, then HCl (2 *M*) was added to the filtrate until the pH value reached 1~2, during which a large amount of precipitate formed. The precipitate was filtered off, washed with distilled water, re-dissolved in KOH solution, and again acidified to pH 1~2. The final acidification product was obtained by filtration and dried *in vacuo* (yield 0.4323 g, 60.3%). ^1^H NMR (300 MHz; DMSO-d^6^): *δ*_H_ 3.31 (3 H, s, CH_3_), 7.03 (2 H, s, ArH), 7.22 (2 H, d, ArH), 7.70 (2 H, d, ArH), 12.70 (2*H*, br, COOH). ^13^C NMR (300 MHz; DMSO-d^6^): *δ*_C_ 40.13 (CH_3_), 115.41 (Ar), 122.24 (Ar), 126.10 (Ar), 128.86 (Ar), 130.52 (Ar), 148.80 (Ar), 167.23 (COOH). MS: *m*/*z* 300.01 [*M*-1]^–^ (Calcd 300.03).

Single crystals of {[Co(C_15_H_9_NO_4_S)(H_2_O)_3_]^.^H_2_O} were obtained by solvothermal reaction of Co(NO_3_)_2_^.^6H_2_O (58.2 mg, 0.2 mmol) and 10-methyl-10*H*-phenothiazine-3,7-dicarboxylic acid (15.6 mg, 0.05 mmol) in a mixed solvent of ethanol and H_2_O (8 ml, volume ratio 1:4) at 393 K for 86 h and finally cooled to room temperature. The resulting products were filtered off, washed thoroughly with distilled water and dried in air at room temperature. The yield was 38.7 mg (45%).

### Refinement

All the H atoms were fixed geometrically and treated as riding with C—H = 0.96 Å, O–H = 0.85 Å and with *U*_iso_ (H) = 1.2*U*_eq_(C) or 1.5*U*_eq_(C, O) for methyl and water H atoms.

### Crystal data

**Table:**

[Co(C_15_H_9_NO_4_S)(H_2_O)_3_]·H_2_O	*F*(000) = 1768
*M**_r_* = 430.30	*D*_x_ = 1.730 Mg m^−^^3^
Orthorhombic, *P**b**c**a*	Mo *K*α radiation, λ = 0.71073 Å
Hall symbol: -P 2ac 2ab	Cell parameters from 8389 reflections
*a* = 15.3105 (8) Å	θ = 2.7–28.1°
*b* = 7.2983 (4) Å	µ = 1.21 mm^−^^1^
*c* = 29.5679 (15) Å	*T* = 291 K
*V* = 3303.9 (3) Å^3^	Block, dark blue
*Z* = 8	0.30 × 0.26 × 0.24 mm

### Data collection

**Table:**

Bruker SMART CCD diffractometer	3236 independent reflections
Radiation source: sealed tube	2650 reflections with *I* > 2σ(*I*)
Graphite monochromator	*R*_int_ = 0.049
φ and ω scans	θ_max_ = 26.0°, θ_min_ = 1.9°
Absorption correction: multi-scan (*SADABS*; Bruker, 2000)	*h* = −18→18
*T*_min_ = 0.703, *T*_max_ = 0.759	*k* = −8→9
16812 measured reflections	*l* = −36→25

### Refinement

**Table:**

Refinement on *F*^2^	Primary atom site location: structure-invariant direct methods
Least-squares matrix: full	Secondary atom site location: difference Fourier map
*R*[*F*^2^ > 2σ(*F*^2^)] = 0.043	Hydrogen site location: inferred from neighbouring sites
*wR*(*F*^2^) = 0.104	H-atom parameters constrained
*S* = 1.08	*w* = 1/[σ^2^(*F*_o_^2^) + (0.0596*P*)^2^] where *P* = (*F*_o_^2^ + 2*F*_c_^2^)/3
3236 reflections	(Δ/σ)_max_ < 0.001
236 parameters	Δρ_max_ = 0.62 e Å^−^^3^
0 restraints	Δρ_min_ = −0.52 e Å^−^^3^

### Special details

**Table:**

Geometry. All e.s.d.'s (except the e.s.d. in the dihedral angle between two l.s. planes) are estimated using the full covariance matrix. The cell e.s.d.'s are taken into account individually in the estimation of e.s.d.'s in distances, angles and torsion angles; correlations between e.s.d.'s in cell parameters are only used when they are defined by crystal symmetry. An approximate (isotropic) treatment of cell e.s.d.'s is used for estimating e.s.d.'s involving l.s. planes.
Refinement. Refinement of *F*^2^ against ALL reflections. The weighted *R*-factor *wR* and goodness of fit *S* are based on *F*^2^, conventional *R*-factors *R* are based on *F*, with *F* set to zero for negative *F*^2^. The threshold expression of *F*^2^ > σ(*F*^2^) is used only for calculating *R*-factors(gt) *etc*. and is not relevant to the choice of reflections for refinement. *R*-factors based on *F*^2^ are statistically about twice as large as those based on *F*, and *R*- factors based on ALL data will be even larger.

### Fractional atomic coordinates and isotropic or equivalent isotropic displacement parameters (Å^2^)

**Table:**

	*x*	*y*	*z*	*U*_iso_*/*U*_eq_	
C1	0.64689 (18)	0.1531 (4)	0.11609 (9)	0.0251 (6)	
C2	0.69797 (18)	0.0972 (4)	0.15690 (8)	0.0226 (6)	
C3	0.65262 (17)	0.0449 (4)	0.19532 (9)	0.0231 (6)	
H3A	0.5899	0.0458	0.1951	0.028*	
C4	0.69635 (17)	−0.0086 (4)	0.23421 (8)	0.0208 (5)	
C5	0.78763 (17)	−0.0105 (4)	0.23556 (8)	0.0186 (5)	
C6	0.83264 (19)	0.0454 (4)	0.19657 (9)	0.0250 (6)	
H6A	0.8953	0.0471	0.1969	0.030*	
C7	0.78842 (19)	0.0987 (4)	0.15843 (9)	0.0256 (6)	
H7A	0.8207	0.1367	0.1323	0.031*	
C8	0.79744 (17)	−0.0200 (4)	0.31735 (8)	0.0193 (5)	
C9	0.70693 (17)	−0.0173 (4)	0.32467 (8)	0.0197 (5)	
C10	0.67305 (17)	0.0274 (4)	0.36651 (9)	0.0211 (5)	
H10A	0.6109	0.0300	0.3708	0.025*	
C11	0.72820 (17)	0.0696 (3)	0.40252 (8)	0.0210 (5)	
C12	0.69238 (17)	0.1164 (4)	0.44740 (8)	0.0208 (5)	
C13	0.81794 (18)	0.0699 (4)	0.39510 (9)	0.0234 (6)	
H13A	0.8563	0.1008	0.4196	0.028*	
C14	0.85212 (17)	0.0271 (4)	0.35350 (9)	0.0230 (6)	
H14A	0.9142	0.0295	0.3491	0.028*	
C15	0.92396 (18)	−0.1018 (4)	0.27125 (10)	0.0302 (6)	
H15A	0.9461	−0.1404	0.3001	0.045*	
H15B	0.9348	−0.1955	0.2492	0.045*	
H15C	0.9527	0.0093	0.2622	0.045*	
Co1	0.63098 (2)	0.19466 (5)	0.523713 (12)	0.02238 (13)	
N1	0.83090 (14)	−0.0684 (3)	0.27475 (7)	0.0212 (5)	
O1	0.74252 (13)	0.1653 (3)	0.47923 (6)	0.0267 (4)	
O2	0.61043 (12)	0.1094 (3)	0.45464 (6)	0.0266 (4)	
O3	0.56576 (13)	0.1431 (3)	0.11768 (7)	0.0363 (5)	
O4	0.68927 (13)	0.2090 (3)	0.08152 (6)	0.0285 (5)	
O5	0.64958 (14)	−0.0747 (3)	0.54390 (8)	0.0359 (5)	
H5X	0.6117	−0.1448	0.5558	0.043*	
H5Y	0.7035	−0.1014	0.5420	0.043*	
O6	0.61132 (15)	0.4686 (3)	0.50278 (7)	0.0356 (5)	
H6X	0.6603	0.5165	0.4962	0.043*	
H6Y	0.5731	0.5414	0.5134	0.043*	
O7	0.50146 (14)	0.1985 (3)	0.54566 (7)	0.0333 (5)	
H7X	0.4691	0.1071	0.5516	0.040*	
H7Y	0.4990	0.2478	0.5717	0.040*	
O8	0.53016 (16)	0.7045 (4)	0.58142 (10)	0.0592 (8)	
H8X	0.5528	0.6002	0.5865	0.071*	
H8Y	0.4840	0.7002	0.5655	0.071*	
S1	0.63643 (4)	−0.08955 (10)	0.28101 (2)	0.02552 (18)	

### Atomic displacement parameters (Å^2^)

**Table:**

	*U*^11^	*U*^22^	*U*^33^	*U*^12^	*U*^13^	*U*^23^
C1	0.0279 (14)	0.0268 (15)	0.0205 (13)	−0.0044 (11)	−0.0003 (11)	0.0048 (11)
C2	0.0303 (14)	0.0213 (13)	0.0163 (13)	0.0022 (11)	0.0007 (10)	0.0014 (10)
C3	0.0219 (13)	0.0276 (14)	0.0199 (14)	−0.0005 (10)	−0.0005 (10)	0.0009 (10)
C4	0.0221 (13)	0.0243 (14)	0.0158 (12)	−0.0015 (10)	0.0014 (9)	0.0006 (10)
C5	0.0233 (13)	0.0200 (13)	0.0126 (12)	0.0005 (10)	−0.0016 (9)	−0.0027 (10)
C6	0.0250 (13)	0.0341 (15)	0.0160 (14)	−0.0021 (11)	0.0010 (10)	−0.0019 (11)
C7	0.0288 (14)	0.0294 (15)	0.0186 (13)	−0.0013 (12)	0.0050 (10)	0.0015 (11)
C8	0.0237 (13)	0.0192 (13)	0.0151 (12)	0.0016 (10)	0.0011 (10)	0.0031 (9)
C9	0.0229 (13)	0.0180 (13)	0.0182 (12)	−0.0023 (10)	−0.0015 (10)	0.0019 (9)
C10	0.0206 (12)	0.0253 (14)	0.0176 (13)	0.0011 (11)	0.0017 (10)	−0.0001 (10)
C11	0.0261 (14)	0.0212 (14)	0.0156 (12)	−0.0004 (10)	0.0016 (10)	0.0009 (10)
C12	0.0271 (13)	0.0194 (13)	0.0160 (12)	0.0010 (10)	0.0018 (10)	−0.0015 (10)
C13	0.0239 (13)	0.0296 (14)	0.0168 (13)	−0.0050 (11)	−0.0028 (10)	−0.0035 (10)
C14	0.0181 (12)	0.0320 (15)	0.0188 (13)	0.0001 (10)	−0.0017 (10)	−0.0007 (11)
C15	0.0241 (14)	0.0416 (18)	0.0250 (14)	0.0104 (12)	−0.0048 (11)	−0.0042 (12)
Co1	0.0213 (2)	0.0297 (2)	0.0162 (2)	−0.00106 (14)	0.00060 (14)	−0.00571 (14)
N1	0.0172 (10)	0.0312 (13)	0.0151 (11)	0.0037 (9)	−0.0023 (8)	−0.0009 (9)
O1	0.0220 (10)	0.0381 (12)	0.0201 (9)	−0.0026 (8)	0.0000 (8)	−0.0081 (8)
O2	0.0229 (9)	0.0386 (12)	0.0183 (10)	0.0004 (8)	0.0013 (7)	−0.0063 (8)
O3	0.0260 (11)	0.0556 (14)	0.0273 (11)	−0.0009 (10)	−0.0025 (9)	0.0148 (10)
O4	0.0304 (11)	0.0395 (13)	0.0156 (9)	−0.0006 (9)	0.0002 (8)	0.0092 (8)
O5	0.0358 (12)	0.0328 (12)	0.0392 (13)	0.0047 (9)	−0.0013 (9)	0.0018 (9)
O6	0.0328 (11)	0.0358 (12)	0.0382 (13)	0.0003 (10)	0.0049 (9)	0.0062 (10)
O7	0.0272 (11)	0.0420 (12)	0.0308 (12)	−0.0065 (9)	0.0057 (9)	−0.0104 (9)
O8	0.0315 (13)	0.0651 (19)	0.081 (2)	−0.0039 (12)	−0.0143 (13)	0.0253 (14)
S1	0.0230 (3)	0.0396 (4)	0.0139 (3)	−0.0101 (3)	−0.0013 (2)	0.0024 (3)

### Geometric parameters (Å, º)

**Table:**

C1—O3	1.245 (3)	C12—O2	1.274 (3)
C1—O4	1.278 (3)	C12—Co1	2.510 (2)
C1—C2	1.495 (4)	C13—C14	1.373 (4)
C2—C3	1.385 (4)	C13—H13A	0.9601
C2—C7	1.386 (4)	C14—H14A	0.9600
C3—C4	1.387 (4)	C15—N1	1.449 (3)
C3—H3A	0.9600	C15—H15A	0.9601
C4—C5	1.398 (4)	C15—H15B	0.9599
C4—S1	1.762 (3)	C15—H15C	0.9600
C5—N1	1.400 (3)	Co1—O4^i^	2.0523 (19)
C5—C6	1.404 (4)	Co1—O5	2.074 (2)
C6—C7	1.371 (4)	Co1—O7	2.087 (2)
C6—H6A	0.9601	Co1—O6	2.114 (2)
C7—H7A	0.9594	Co1—O2	2.1581 (19)
C8—C14	1.400 (4)	Co1—O1	2.1661 (19)
C8—C9	1.403 (4)	O4—Co1^ii^	2.0523 (19)
C8—N1	1.405 (3)	O5—H5X	0.8497
C9—C10	1.381 (4)	O5—H5Y	0.8500
C9—S1	1.763 (3)	O6—H6X	0.8499
C10—C11	1.393 (4)	O6—H6Y	0.8500
C10—H10A	0.9601	O7—H7X	0.8500
C11—C13	1.391 (4)	O7—H7Y	0.8500
C11—C12	1.476 (3)	O8—H8X	0.8500
C12—O1	1.266 (3)	O8—H8Y	0.8499
			
O3—C1—O4	123.7 (3)	N1—C15—H15A	109.5
O3—C1—C2	118.4 (2)	N1—C15—H15B	109.8
O4—C1—C2	117.9 (2)	H15A—C15—H15B	109.5
C3—C2—C7	118.5 (2)	N1—C15—H15C	109.2
C3—C2—C1	118.4 (2)	H15A—C15—H15C	109.5
C7—C2—C1	123.2 (2)	H15B—C15—H15C	109.5
C2—C3—C4	121.0 (2)	O4^i^—Co1—O5	91.44 (9)
C2—C3—H3A	119.7	O4^i^—Co1—O7	98.60 (8)
C4—C3—H3A	119.3	O5—Co1—O7	93.09 (9)
C3—C4—C5	120.6 (2)	O4^i^—Co1—O6	88.96 (9)
C3—C4—S1	119.6 (2)	O5—Co1—O6	179.58 (10)
C5—C4—S1	119.6 (2)	O7—Co1—O6	86.73 (9)
C4—C5—N1	120.0 (2)	O4^i^—Co1—O2	161.86 (8)
C4—C5—C6	117.7 (2)	O5—Co1—O2	91.09 (9)
N1—C5—C6	122.4 (2)	O7—Co1—O2	99.19 (8)
C7—C6—C5	121.0 (3)	O6—Co1—O2	88.56 (8)
C7—C6—H6A	119.9	O4^i^—Co1—O1	101.35 (8)
C5—C6—H6A	119.1	O5—Co1—O1	88.44 (8)
C6—C7—C2	121.2 (3)	O7—Co1—O1	159.95 (8)
C6—C7—H7A	119.4	O6—Co1—O1	91.61 (8)
C2—C7—H7A	119.4	O2—Co1—O1	60.78 (7)
C14—C8—C9	118.0 (2)	O4^i^—Co1—C12	131.59 (8)
C14—C8—N1	121.9 (2)	O5—Co1—C12	89.52 (9)
C9—C8—N1	120.1 (2)	O7—Co1—C12	129.68 (9)
C10—C9—C8	120.8 (2)	O6—Co1—C12	90.31 (9)
C10—C9—S1	119.8 (2)	O2—Co1—C12	30.49 (8)
C8—C9—S1	119.19 (19)	O1—Co1—C12	30.29 (8)
C9—C10—C11	120.6 (2)	C5—N1—C8	119.6 (2)
C9—C10—H10A	119.7	C5—N1—C15	117.2 (2)
C11—C10—H10A	119.6	C8—N1—C15	117.7 (2)
C13—C11—C10	118.6 (2)	C12—O1—Co1	90.08 (16)
C13—C11—C12	120.6 (2)	C12—O2—Co1	90.23 (15)
C10—C11—C12	120.9 (2)	C1—O4—Co1^ii^	123.71 (18)
O1—C12—O2	118.9 (2)	Co1—O5—H5X	126.6
O1—C12—C11	120.6 (2)	Co1—O5—H5Y	109.4
O2—C12—C11	120.5 (2)	H5X—O5—H5Y	123.5
O1—C12—Co1	59.64 (13)	Co1—O6—H6X	109.3
O2—C12—Co1	59.28 (13)	Co1—O6—H6Y	125.5
C11—C12—Co1	179.7 (2)	H6X—O6—H6Y	115.7
C14—C13—C11	121.2 (2)	Co1—O7—H7X	127.4
C14—C13—H13A	119.8	Co1—O7—H7Y	109.3
C11—C13—H13A	119.1	H7X—O7—H7Y	96.8
C13—C14—C8	120.8 (2)	H8X—O8—H8Y	113.8
C13—C14—H14A	119.7	C4—S1—C9	98.98 (12)
C8—C14—H14A	119.5		
			
O3—C1—C2—C3	−2.9 (4)	O1—C12—Co1—O5	−87.73 (16)
O4—C1—C2—C3	176.9 (3)	O2—C12—Co1—O5	92.97 (16)
O3—C1—C2—C7	178.4 (3)	O1—C12—Co1—O7	178.66 (15)
O4—C1—C2—C7	−1.8 (4)	O2—C12—Co1—O7	−0.6 (2)
C7—C2—C3—C4	−1.4 (4)	O1—C12—Co1—O6	92.65 (16)
C1—C2—C3—C4	179.9 (3)	O2—C12—Co1—O6	−86.64 (16)
C2—C3—C4—C5	0.2 (4)	O1—C12—Co1—O2	179.3 (3)
C2—C3—C4—S1	−175.5 (2)	O2—C12—Co1—O1	−179.3 (3)
C3—C4—C5—N1	−178.7 (2)	C4—C5—N1—C8	−38.7 (4)
S1—C4—C5—N1	−3.0 (3)	C6—C5—N1—C8	141.9 (3)
C3—C4—C5—C6	0.7 (4)	C4—C5—N1—C15	168.1 (2)
S1—C4—C5—C6	176.5 (2)	C6—C5—N1—C15	−11.3 (4)
C4—C5—C6—C7	−0.5 (4)	C14—C8—N1—C5	−141.7 (3)
N1—C5—C6—C7	178.9 (3)	C9—C8—N1—C5	38.1 (4)
C5—C6—C7—C2	−0.7 (4)	C14—C8—N1—C15	11.3 (4)
C3—C2—C7—C6	1.6 (4)	C9—C8—N1—C15	−168.9 (2)
C1—C2—C7—C6	−179.7 (3)	O2—C12—O1—Co1	0.7 (3)
C14—C8—C9—C10	−1.1 (4)	C11—C12—O1—Co1	−179.8 (2)
N1—C8—C9—C10	179.1 (2)	O4^i^—Co1—O1—C12	−177.13 (16)
C14—C8—C9—S1	−176.1 (2)	O5—Co1—O1—C12	91.71 (17)
N1—C8—C9—S1	4.1 (3)	O7—Co1—O1—C12	−3.0 (3)
C8—C9—C10—C11	−0.5 (4)	O6—Co1—O1—C12	−87.87 (17)
S1—C9—C10—C11	174.5 (2)	O2—Co1—O1—C12	−0.41 (15)
C9—C10—C11—C13	1.5 (4)	O1—C12—O2—Co1	−0.7 (3)
C9—C10—C11—C12	−179.6 (2)	C11—C12—O2—Co1	179.8 (2)
C13—C11—C12—O1	3.2 (4)	O4^i^—Co1—O2—C12	10.8 (4)
C10—C11—C12—O1	−175.7 (3)	O5—Co1—O2—C12	−87.19 (16)
C13—C11—C12—O2	−177.3 (3)	O7—Co1—O2—C12	179.51 (16)
C10—C11—C12—O2	3.8 (4)	O6—Co1—O2—C12	93.05 (16)
C10—C11—C13—C14	−0.9 (4)	O1—Co1—O2—C12	0.41 (15)
C12—C11—C13—C14	−179.9 (3)	O3—C1—O4—Co1^ii^	3.4 (4)
C11—C13—C14—C8	−0.6 (4)	C2—C1—O4—Co1^ii^	−176.40 (18)
C9—C8—C14—C13	1.7 (4)	C3—C4—S1—C9	−149.7 (2)
N1—C8—C14—C13	−178.5 (3)	C5—C4—S1—C9	34.6 (2)
O1—C12—Co1—O4^i^	3.8 (2)	C10—C9—S1—C4	150.0 (2)
O2—C12—Co1—O4^i^	−175.52 (15)	C8—C9—S1—C4	−35.0 (2)

Symmetry codes: (i) *x*, −*y*+1/2, *z*+1/2; (ii) *x*, −*y*+1/2, *z*−1/2.

### Hydrogen-bond geometry (Å, º)

**Table:**

*D*—H···*A*	*D*—H	H···*A*	*D*···*A*	*D*—H···*A*
O5—H5*X*···O8^iii^	0.85	1.83	2.678 (4)	180
O5—H5*Y*···O4^iv^	0.85	2.16	2.879 (3)	142
O6—H6*X*···O1^v^	0.85	1.91	2.748 (3)	169
O6—H6*Y*···O8	0.85	2.43	3.149 (4)	143
O7—H7*X*···O2^vi^	0.85	2.00	2.826 (3)	163
O7—H7*Y*···O3^i^	0.85	1.88	2.615 (3)	144
O8—H8*X*···O3^i^	0.85	2.01	2.808 (4)	156
O8—H8*Y*···O2^vii^	0.85	2.09	2.760 (3)	135

Symmetry codes: (i) *x*, −*y*+1/2, *z*+1/2; (iii) *x*, *y*−1, *z*; (iv) −*x*+3/2, −*y*, *z*+1/2; (v) −*x*+3/2, *y*+1/2, *z*; (vi) −*x*+1, −*y*, −*z*+1; (vii) −*x*+1, −*y*+1, −*z*+1.
